# A Novel Spider-Inspired Rotary-Rolling Diaphragm Actuator with Linear Torque Characteristic and High Mechanical Efficiency

**DOI:** 10.1089/soro.2020.0108

**Published:** 2022-04-19

**Authors:** Jonas Hepp, Alexander Badri-Spröwitz

**Affiliations:** Dynamic Locomotion Group, Max Planck Institute for Intelligent Systems, Stuttgart, Germany.

**Keywords:** rotary, rolling, diaphragm, actuator, efficient, spider-inspired, fluidic, MRI compatible

## Abstract

We present a novel, fluid-driven rotary-rolling diaphragm actuator with direct rotary output. Its working principle is inspired by the spider leg's hydraulically operated joints and the diaphragm design of rolling diaphragm actuators. The new actuator is fully sealed, shows minimal output torque losses, and minimum friction during operation. Stiction and Coulomb friction are avoided by design. Our proposed mechanism can be used as a compliant actuator in soft robots, or as a stiff transmission device, depending on the fluid and working pressure. The rotary-rolling diaphragm is the defining component of the actuator. The diaphragm is based on silicone rubber, reinforced by a fabric with anisotropic tensile strength characteristics. The diaphragm is custom-designed to follow the actuator's toroidal shape and to ensure the smooth unrolling behavior throughout the stroke. Our actuator outputs a constant torque throughout its stroke compared with monolithic, rotary soft robot actuators with a change in torque. Our design offers a high mechanical efficiency of 95%, compactness, a wide working range of 100°, and a low mechanical complexity from a single chamber.

## Introduction

Fluidic actuators are omnipresent; they are installed as miniature actuators^1^ or in giant excavators, and in mobile and energy-limited legged robots.^2^ The compact and power-dense fluidic actuation principle allows power distribution and gearing from a central accumulator to distal actuators, with lightweight moving parts. Piston–cylinders as telescopic fluidic actuators output a constant force at isobaric conditions, linear in relation to pressure. But external mechanisms are required to convert forces to torque, resulting in efficiency losses, nonlinear force characteristics,^3^ and additional complexity.

**FIG. 1.**
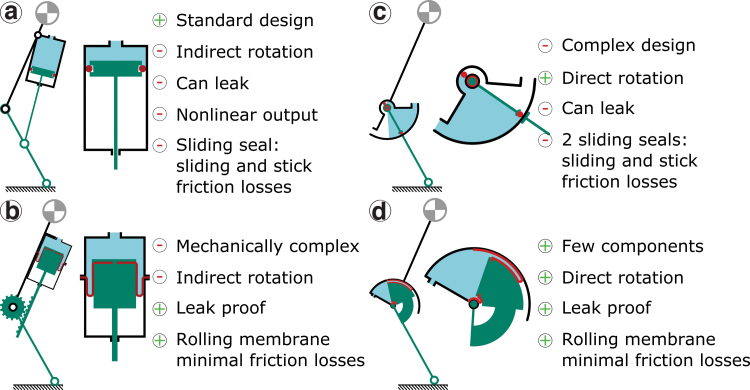
Design methodology leading to the rotary-rolling diaphragm actuator requirements. **(a)** Conventional fluidic actuators. They can achieve high force efficiency. The O-ring as the seal between the cylinder and the piston leads to Coulomb friction and stiction. **(b)** Rolling diaphragm actuators avoid Coulomb and stick friction by a diaphragm rolling between walls. They output telescopic movement. **(c)** Vane actuators directly output torque but require two seals with high mounting and manufacturing precision, and a complex shape. **(d)** We propose a rotary-rolling diaphragm mechanism that uses the rolling diaphragm concept, but for direct torque output. The actuator can be manufactured with the mere help of a 3D printer and manual tools. It is important to note that the pairing of the pinion/rack and direct linkage system with the O-ring or rolling diaphragm cylinder is arbitrary and could be switched. In this case, the points “nonlinear output” and “mechanically complex” would switch. 3D, three-dimensional. Color images are available online.

Alternatively, vane actuators directly output torque.^4^ Piston–cylinder and vane actuators carry press-fit seals, leading to stiction and Coulomb friction.^5^ The mechanical work efficiency of piston–cylinder actuators can be as high as 95% especially in the high force output range, but is typically 80–90%.^5,6^ Two seals are mounted in vane actuators (Fig. 1c),^7,8^ reducing efficiency to around 80%.^7^

Rolling diaphragm actuators are fluidic actuators with a soft and bendable rolling diaphragm, no significant Coulomb friction or stiction.^9^ However, they are only available as telescopic actuators. Classic soft pneumatic actuators (SPAs) are designed for telescopic or rotary displacement. SPAs are made from stretchable elastomer membranes or fiber-reinforced elastomer, creating “bladders” or “diaphragms.”^10–12^ These designs are robust, simple, leakproof, and require no seals.^13^ But bladder designs show a decrease in output force during isobaric operation, caused by membrane internal forces.^10,14,15^ Consequently, viscoelastic membrane deformation should be minimized for mechanical efficiency. Further losses arise from uncontrolled and unwanted bladder deformations.^16^

Pneumatic artificial muscles (PAMs) such as McKibben muscles limit bladder deformations with a membrane internal mesh of reinforcing fibers. PAMs suffer from large, nonelastic membrane deformations caused by their rubber-like material, which reduces actuator efficiency and introduces a nonlinear pressure–force relationship.^17^ Soft fluidic/pneumatic robots are versatile, but only 25–49% mechanically efficient.^14,18^ In mobile and dynamic applications, a low actuator efficiency is prohibitive: energy consumption increases, and output force and speed decrease.

**FIG. 2.**
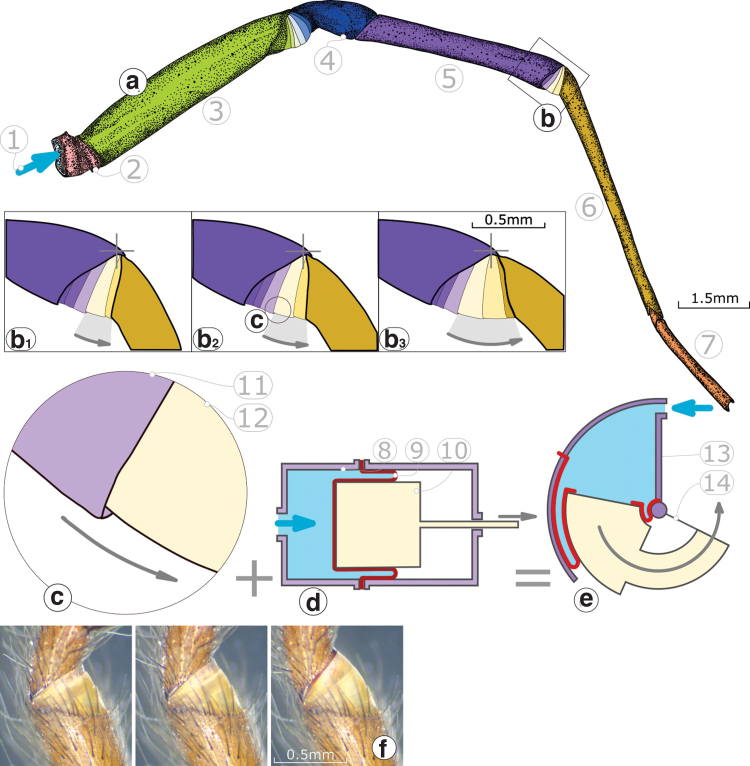
Simplified view **(a)** of a common house spider leg (genus *Tegenaria*), with a close look at the membrane of the tibia–metatarsus joint **(b, b1–b3)**. Several arc-shaped **(c)**, stacked membrane segments unfold successively at joint opening. (1) Pressurized fluid inflow, (2) coxa and trochanter, (3) femur, (4) patella, (5) tibia, (6) metatarsus, (7) tarsus. **(d)** A linear fluidic actuator based on a rolling diaphragm. In our work, we combine the idea of a rolling diaphragm with the stacked membrane segments of the spider's joint, into **(e)** a rotary-rolling diaphragm actuator. (8) Housing, (9) rolling diaphragm, (10) piston, (11) and (12) membrane segments, (13) housing, (14) rotating piston. **(f)** Microscope images of the tibia–metatarsus joint, with its schematic presentation in **(b)**. Color images are available online.

Fluidic actuation evolved in animals and plants.^19–23^ In the presented work, we take inspiration from the membrane structure found in the femur–patella and tibia–metatarsus joints of spiders (Fig. 2b). They are hypothesized to open hydraulically by pressurized hemolymph^24^ and show an “arthrodial” or “articular” membrane (Fig. 2f), possibly formed from a single “folded” membrane.^24–26^

The hypothesis that hydraulic work extends joints is still discussed,^22,27^ but inspired many robots and applications.^28–30^ A spider-inspired leg joint included a pressurized plastic bag, with good static torque characteristics, but dynamic losses.^31^ A pneumatically actuated finger joint with bellow-like joint structures provides intrinsic compliance during manipulation.^32^ The spider's hemolymph-actuated leg joints were role models for “Athrobot.”^16^ Its inflatable, balloon-like actuators are miniature-sized, react within 100 ms, but show substantial losses caused by work required to strain the elastomer material.

For the presented design of a spider-inspired fluidic actuator with a soft rolling diaphragm, we adopt the hypothesis that (1) spider leg joints are opened from pressurized fluid, and (2) the membrane structure acts as limit for the effective lever arm and active pressure area to (3) produce joint output torque and work. We propose a new rotary actuator that avoids Coulomb friction and stiction, external gearing, and viscoelastic deformation. To achieve this, we combine the rolling diaphragm actuator concept with the membrane morphology observed in spider joints (Fig. 2f). Our work focuses on the material, shape, and fabrication of a new rotary-rolling diaphragm actuator, and we characterize the actuator's output torque and friction to output work efficiency.

## Methods

We initially explain our design methodology leading to the rotary-rolling diaphragm actuator, and the actuator's design and implementation process. We describe the experimental setup for quantifying the actuator's output characteristics, under isometric and dynamic (quasi-isobaric) conditions.

### Design methodology

Piston–cylinder actuators are standardized products, driven hydraulically or pneumatically (Fig. 1). O-rings mounted on the pistons act as a fluid barrier, but lead to Coulomb friction and stiction.^33,34^ Mounting telescopic actuators between leg segments leads to a nonlinear force profile (Fig. 1a).^3^ External mechanics convert telescopic into rotation (Fig. 1b), but with extra complexity and efficiency loss. Industrial piston–cylinder actuators reach mechanical efficiencies up to 95%,^5^ and designs without O-ring s even 99%, but at limited working pressures.^35^ Vane actuators directly output torque but require two seals, leading to a mechanical efficiency between 70% and 80% (Fig. 1c).^7,8^

The tube-shaped diaphragms of rolling diaphragm actuators unroll around their active edge, with the rest of the diaphragm laying flat against the piston and cylinder wall (Fig. 1b). The diaphragm rolls without Coulomb friction or stiction, and with force losses as low as 2–5%.^36^ The diaphragm is slightly strechable perpendicular to the rolling direction.^37^ The rolling edge $d_{gap}$ withstands the tensile stress σ from fluid pressure *p*: $\sigma =\frac{1}{2}pd_{gap}.$^38^ To achieve both, commercial diaphragms are made from fabric reinforced elastomers for tensile strength and anisotropy, while being flexible and fluid-impermeable.^39^ But a positive pressure-differential is required, otherwise the diaphragm forms creases and jams the piston.^40^

The characteristics of SPA robots are largely “hard-coded” in material and morphology, with unique locomotion capabilities.^10,13,41,42^ But soft actuators and their robots can be hard to control and show low mechanical efficiency; stretching the bladder changes its shape, and parasitic volumes form without contributing to external network. A change of shape also alters the torque-active pressure area and the lever arm length. The material's elasticity and viscosity produce membrane-internal forces, which oppose the working direction. Finally, elastomers exhibit hysteresis and memory effect losses.^43,44^ As a result, the output torque and efficiency of rotary soft actuators depend on their working angle. The membrane expansion can be guided by shells, but the expanding bladder will slide against these.^15,31,45^

We conclude that rolling diaphragms have the best potential for high-efficiency actuators. They produce minimal viscoelastic stretch, almost no parasitic volumes, and avoid stiction and Coulomb friction. So far, rolling diaphragm actuators were limited to telescopic action, and external mechanisms were required to produce torque.^9,46^

### Diaphragm and actuator implementation

**FIG. 4.**
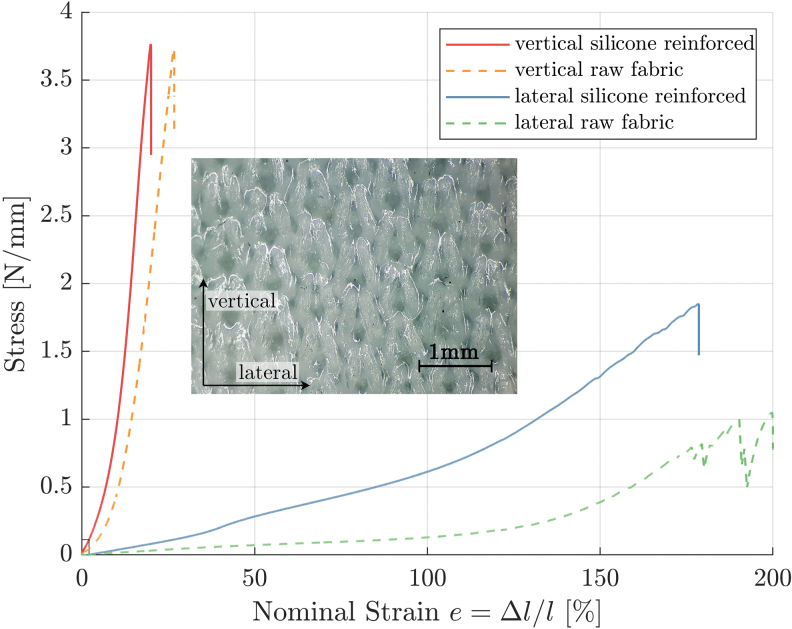
Uniaxial stress–strain characteristics of the fabric core and the resulting diaphragm material. Embedded is a microscope image of the diaphragm. Stress is shown in [N/mm] instead of [Pa]. The thickness of the diaphragm (0.5 mm) is largely determined by its fabric core thickness (0.35 mm). The rotary-rolling diaphragm here is stressed to maximum values barely visible in this plot, around 0.11 N/mm, indicated by the small rectangle at the plot's origin. Hence, by replacing the 3D printed plastic with a stiffer material, the actuator's maximum working pressure can be increased. Color images are available online.

The diaphragm of our rotary-rolling diaphragm actuator has a fabric core with anisotropic elastic properties.^47^ The fabric stretches 26 × more easily in one, compared with the perpendicular direction, due to its knitting pattern. We soaked and coated the textile in silicone rubber, which increases the stiffness of the cured diaphragm in both directions yet retains its anisotropic properties (Fig. 4). These anisotropic properties are essential, as the diaphragm needs to be flexible in the lateral direction, to be able to adapt to the new diameter when unrolling. At the same time, it needs to be stiff in the vertical direction, to minimize losses from undesired deformation of the rolled edge.^37,38^

As a rotary actuator, the diaphragm requires a more complex, asymmetrical shape conforming to the shell part, compared with the cylindrical shape of a standard rolling diaphragm (Fig. 1b). Only a diaphragm shape designed for minimal internal stress unrolls smoothly and with high efficiency.

**FIG. 3.**
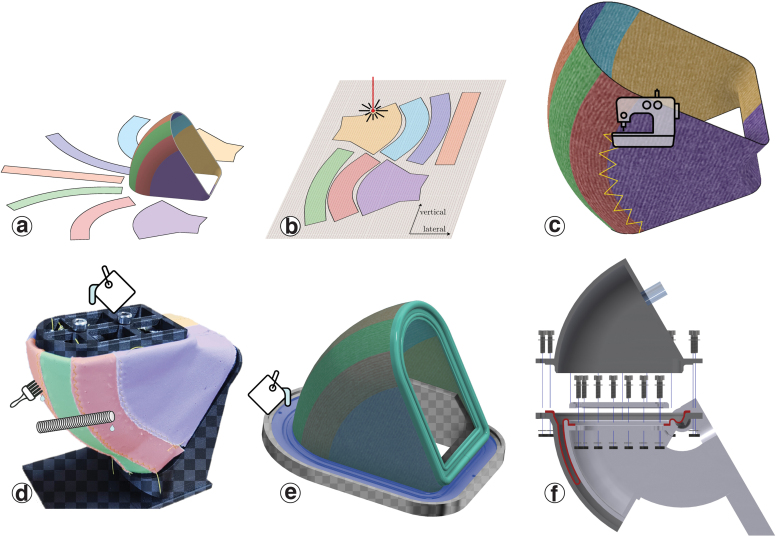
Diaphragm fabrication process. The diaphragm's core is sewn together from seven fabric patches and coated with silicone rubber. Seams run in parallel to the rolling direction. **(a)** The patch is shaped by dividing the piston's surface into seven segments, unrolling the segments into 2D, and adding margins for sewing and clamping. **(b)** Patches are cut from a microfiber cloth with a laser cutter. **(c)** Patches are sewn together with a sewing machine. **(d)** The diaphragm core fabric is placed over a positive form for coating, defining its final shape, and then manually soaked with liquid silicone. Excessive rubber is brushed off, and the upper rim (mold included) is cast immediately. The rim will clamp down the diaphragm in the actuator. **(e)** The cured and coated diaphragm is placed into a second mold to cast the lower rim. **(f)** Assembly: the diaphragm is clamped to the piston and then between the two shell halves. Color images are available online.

The designs starts with the shell and its piston (Fig. 3a). The piston walls and the diaphragm are toroidally shaped. The diaphragm's fabric core consists of seven individual patches. Their shapes were generated by parting the piston's surface into seven pieces, with a computer-aided design (CAD) program. We unrolled the pieces with minimal distortion into two-dimensional (2D) (Autodesk Meshmixer), added a 5 mm margin to all outlines as sewing overlap, and 2 cm at the top and bottom for clamping. The patches were then laser cut (PLS 6.150D) from a microfiber cloth with anisotropic stretch characteristics (Fig. 4). We used a microfiber cleaning cloth (Stratasys, part number MSC-00014-S), but we observed similar properties in other commonly found microfiber cleaning cloths with anisotropic stretch characteristics. The seven patches were sewn together on a sewing machine (Toyota SuperJ15), with a stretchable “zig-zag” stitch. Stitching creates slightly protruding seams. For a smooth diaphragm unrolling throughout the stroke, seams were planned parallel to the actuator's perimeter, perpendicular to the rolling edge (Fig. 3).

To avoid distortions from flattening and reshaping, we treated and cured the fabric in its three-dimensional (3D) shape, by mounting it over a positive form. The fabric was soaked and covered with silicone rubber (Ecoflex 50), excess material was brushed off with a hand coating tool, for a uniform film thickness. The fabric and the resulting diaphragm are 0.35 and 0.5 mm thick, respectively.

We added rims made from a stiffer silicone rubber (Dragon Skin 30), both on the upper and the lower edge of the diaphragm (Fig. 3d, e). They ease the assembly and allow for a good seal. The upper rim was cast during the fabric's coating (Fig. 3d), and the lower rim after curing the diaphragm.

**FIG. 5.**
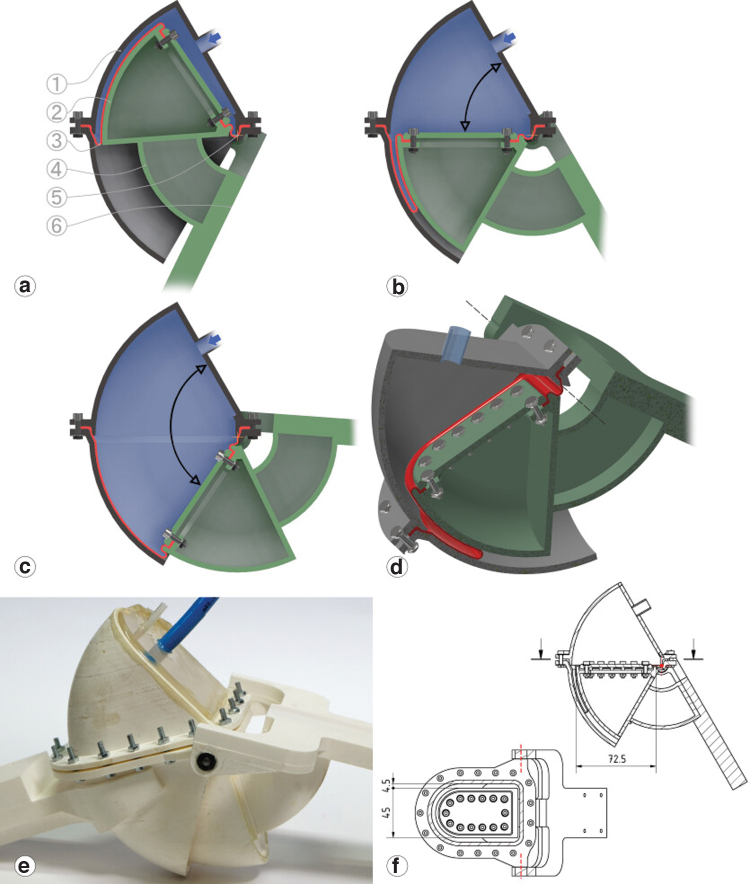
**(a–d)** Opening sequence, cut view of the rotary-rolling diaphragm actuator. (1) Shell (*gray*), (2) piston (*green*), (3) diaphragm (*red*), (4) bridge (*green*), (5) axis, mounted outside the shell, (6) torque is applied to the moving leg segment (*green*). The diaphragm is shown in *red*, the pressurized fluid volume in *blue*. When the piston moves, the outer diaphragm portion covers the largest distance and thus unrolls rapidly. Here, a smooth and low-friction behavior is essential. Shell and piston are split into two parts connected by screws clamping the diaphragm. Shell and piston are 3D-printed from ABS; the base of the shell is closed by a transparent polymethylmethacrylate plate. The *blue hose* is the supply hose for pressurized air and connects to a valve. The smaller transparent hose connects to the pressure sensor. ABS, acrylonitrile butadiene styrene. Color images are available online.

The shell and its piston are toroidal surfaces of rotation (Fig. 5f). The piston requires a hinge joint to enforce a rotary motion and convert the fluid pressure into torque. An axis directly connecting the piston and the shell would need to pass through the diaphragm, puncturing it. We extended the piston by a bridge instead (Fig. 5d, e). Our design allows a working range of 100°, theoretically up to 180°. Both the shell and the piston are assembled from two parts, screwed together, and the diaphragm's inner and outer rims are clamped in-between. The actuator weights in total 384 g, including pieces to connect it to the test-stand (Table 2).

**Table 2.** Weight of Parts Derived from the Computer-Aided Design Model

Part	Weight [g]
Piston	48
Shell	111
Bridge + connector to axis	34
Axis	12
31 × screws, nuts	65
Piston test-stand mount	49
Shell test-stand mount	57
Diaphragm	8
Total	384

The piston, shell, bridge, and test-stand mounts are all three-dimensional printed from standard acrylonitrile butadiene styrene (ABS). The axis and screws used are made of metal but could also be made of carbon fiber or plastic. The diaphragm's weight is calculated based on the diaphragm area and thickness, assuming a silicone rubber density of 1.07 g/cm^−3^. The above shown actuator weight would not be representative for an optimized system; the shown items are part of the experimental setup, for a nonoptimized proof-of-concept system.

### Experimental setup

**FIG. 6.**
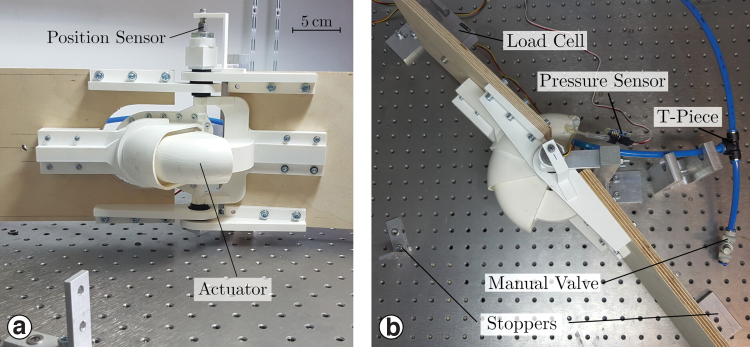
**(a)** *Side view* and **(b)** *Top view* of the test-stand. The test-stand is composed of two arms connected to the piston and the shell, respectively. The shell connects to the *left arm*, fixed to the ground via a load cell to measure the torque. A position sensor records the opening angle. A pressure sensor measures chamber pressure and is connected by a second, *short hose*. The pressure source and regulator are wall-mounted and not shown here. Color images are available online.

We characterized the actuator's output torque and friction in a test-stand. The actuator is mounted onto two arms on an optical table (Fig. 6). One arm is fixed against a load cell (Tedea-Huntleigh 1042) with a lever arm of 22.3 mm, to measure the actuator's output force and calculate the torque. Actuator pressure is measured with a sensor (Phidget 1140) connected by a short separate tube. The joint angle is measured with a potentiometer (Vishay ECS 78). A converter connected via USB to a PC (NI-USB 6343; National Instruments) samples all signals at 400 Hz. Data is recorded and processed in Matlab.

### Preparation of isometric experiments

During isometric experiments, the piston arm was fixed in selected joint positions. Air pressure was applied to the actuator at each position, and torque–pressure were measured. The actuator's torque was calculated as:

(1) M=pAr,

where *A* is the active area of the piston. The exact pressure effective area will be determined experimentally (see the Isometric Experiments section; Table 1). Initially, the piston's base area (3044 mm^2^) is used. *r* is the lever arm of the geometric center of the area (39 mm), and *p* is the difference between chamber and atmospheric pressure (*p* = 0–50 kPa).

**Table 1.** Estimated and Measured Actuator Output Torque, at 50 kPa Chamber Pressure

M/A	Active components	Area [mm^2^]	Lever arm [mm]	Ar [m^3^ × 10^−4^]	Torque = pAr at 50 kPa [Nm]
Model 1	Piston base	3044	39.0	1.19	5.9
Model 2	Piston base + full gap	4070	38.8	1.58	7.9
Model 3	Piston base + 0.5 gap	3557	38.9	1.38	6.9
Actuator	Measured	—	—	1.46	7.3

The output torque depends on the effective piston area and lever arm, that is, the sum of all area *A* perpendicular to the rotation direction, and its lever arm *r*. The measured actuator's output torque lies just above the torque of the model “piston base + 0.5 gap.”

### Preparation of work-loop experiments

In the work-loop experiments, the actuator's friction losses during movement are characterized, and its mechanical work efficiency is established. Frictional losses act against the direction of motion. During actuator closing, friction adds a torque ($M_{friction}$) to the intended actuator torque [Eq. (1)], which both need to be overcome to close the actuator, leading to an observed torque:

(2) $$M_{closing}=pAr+M_{friction}.$$

A similar friction torque component acts during actuator opening, this time opposing the actuator opening torque and thus reducing the measured torque:

(3) $$M_{opening}=pAr-M_{friction}.$$

If the chamber pressure is constant (“isobaric”), then the hysteresis area from the positive and negative shift around the working torque allows quantifying the energy lost per cycle. This further allows calculating a mean friction torque for each cycle. Potential spring-like forces would have no impact since they would shift both measured torques upward or downward without affecting the area in-between. Perfectly isobaric conditions would allow to calculate friction losses directly. For practical reasons, we can only establish “equivalent” isobaric conditions; first, by the mechanical setup, and then, by a numerical compensation for the remaining pressure deviation.

Air is supplied by an in-house pressure line, manually pressure limited. A T-piece inputs the air pressure to the actuator and also connectors a manual valve leading to the environment (Fig. 6). We set target pressures by adjusting the regulator and the valve's output flow. At steady-state operation, a constant amount of air leaves the valve. While the actuator opens and its internal volume increases, additional air is supplied by the pressure regulator, and less air escapes through the T-connector.

**FIG. 7.**
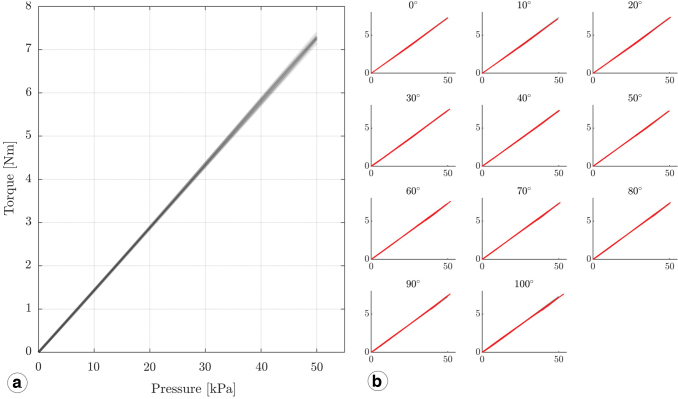
Isometric experiments: both actuator arms are immobile, and joint torque is measured. The actuator's output torque is shown, for chamber pressures from 0 to 50 kPa and joint angles from 0° to 100°. **(a)** Eleven curves are overlaid, each smoothed with a fourth-order polynomial fit. The maximum isometric actuator torque measured is 7.3 Nm. **(b)** For each joint angle position from 0° to 100°, the chamber pressure was slowly increased to 50 kPa and then slowly decreased again. Color images are available online.

**FIG. 8.**
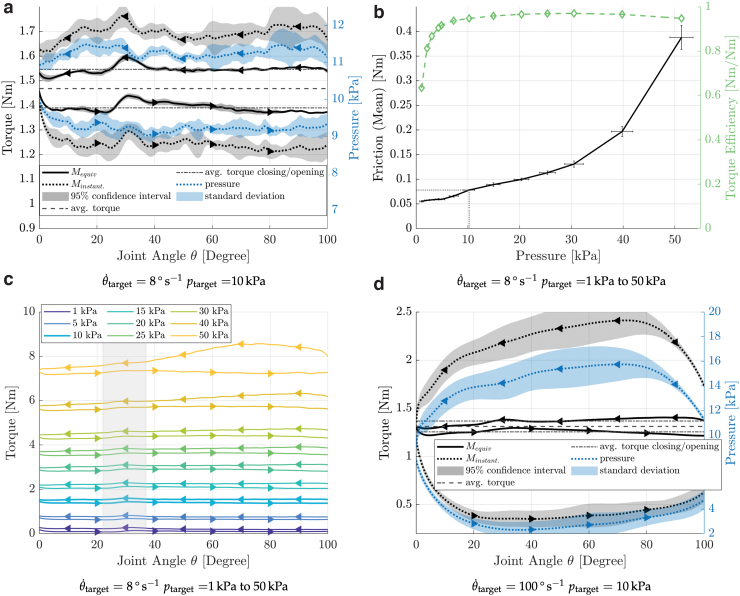
Experimental results from work-loop experiments. **(a)** Torque and pressure curves for an average pressure of 10.25 kPa (target pressure of 10 kPa) and joint speed of 8°/s. The graph shows five consecutive measurements, merged. The upper curves represent values at joint closing, the lower at joint opening. The pressure at joint closing is higher than at opening since additional air has to flow out of the actuator. The torque shown is corrected for the difference caused by the deviation of the instantaneous pressure from average pressure [M_equiv_, Eq. (5)] using the value established in the static experiments (Ar = 1.46 × 10 − 4 m^3^). The equivalent (“corrected”) torque at joint closing is higher than that at the opening, due to friction. The area enclosed by the two torque curves represents the energy lost due to friction. The bump at 30° is caused by a small spot of additional silicone rubber in that area of the diaphragm. The uncorrected torque curve shows larger fluctuations (*a gray area*), which follows the pressure fluctuations (*blue area*). Here, the instantaneous pressure is not identical between experiments. However, after correcting for pressure fluctuation, the torque curve's uncertainty is small, which confirms that pressure compensation works as intended and indicates that the actuator's torque–friction relationship is repeatable. **(b)** The mean friction and the ratio of friction to nominal torque dependent on the operating pressure. The *error bars* in Y-direction represent the 95% confidence interval of the mean value computed from five measurement cycles. The *error bars* in X-direction represent the mean deviation of the pressure from the mean pressure during the individual measurements. For low pressures up to 25 kPa, the friction slowly and almost linearly increases; for higher pressures, the increase is steeper. The maximum torque efficiency of 97% is at 30 kPa. **(c)** Work-loop experiments, at a joint speed of 8°/s and recalculated torque curves, for pressure levels between 1 and 50 kPa. The *upper curve* of a pair represents the value while closing the joint, the lower while opening. The effect of the piston bending outward at high pressures (Fig. 9) can be seen in the increase of torque difference, for high-pressure scenarios (i.e., 50 kPa), and opening angles above 40°. **(d)** Like **(a)**, but at 100°/s and 10 kPa. Color images are available online.

Although using the pressure regulator and the valve, we still observed a chamber pressure change during joint opening and closing, especially at high joint speed (Fig. 8a, d). We identified the following causes: (1) The actuator was driven manually, and the joint angle speed was not constant, for example, 8°s^−1^ ± 1.8°s^−1^, including the movement's start and stop. (2) Rapid joint movement changes the chamber volume in short time, yet the valve was adjusted for constant airflow with a target pressure at stillstand. The flow changes lead to a mismatch between target and instantaneous pressure. The chamber pressure, joint angle position, and output torque were recorded at all times. To approximate the isobaric conditions, we used the previously recorded linear torque–pressure curve (isometric experiments, Fig. 7). We calculate torques at “equivalent isobaric” conditions as equivalent output torques:

**FIG. 9.**
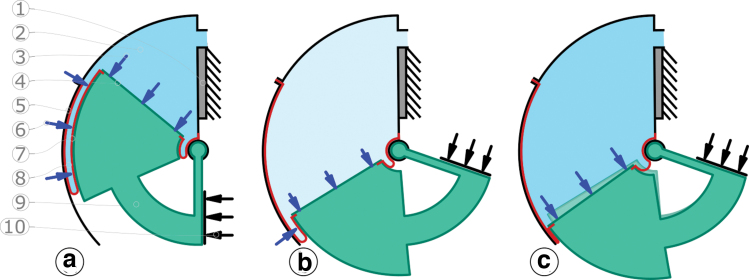
Deformation of the bridge (9) caused by high chamber pressures (*dark blue fluid*) and large opening angles **(c)**. (1) and (10) are stoppers. **(a)** The chamber pressure (2) applies force (*blue arrows*) to the piston area (4), measured by a force sensor at (1). The piston (4) and its bridge (9) are ABS printed. They are curved, and the force pushes the piston radially outward, deforming them elastically **(c)**. While the piston is inserted in the shell (5), the gap's fluid pressure produces a force pushing the piston/bridge toward the center. The effective (net) force (6) depends on the chamber pressure (1), and the pressurized perimeter area (7), that is, the actuator's opening angle. At small actuator angles, the fluid force along the perimeter compensates for the unwanted piston deflection. At combinations of large actuator angle **(b)** and high chamber pressure **(c)**, the piston's bridge deflects eventually outward, closing the outer rotary-rolling diaphragm gap. Even before both diaphragm surfaces contact each other, friction increases: the diaphragm unrolls with a less than optimal gap size. Color images are available online.

(4) $$M_{equiv}=M_{instant.}+\Delta M.$$

With Eq. (1), the difference Δp between average and instantaneous chamber pressure leads to:

(5) ΔM=ΔpAr.

The value for *Ar* will be determined in the Isometric Experiments section. We recorded 2 joint speed sets: $\dot{\theta }=8^{\circ }s^{-1}$ with 13 target chamber pressures (1 … 50 kPa, Fig. 8b) and $\dot{\theta }=100^{\circ }s^{-1}$ with 1 target chamber pressure (10 kPa, Fig. 8d). At least five experimental cycles were averaged for every target pressure. Overall, more than 400 instrumented and noninstrumented experiments were conducted, with no signs of wear on the diaphragm, or the remaining actuator.

## Results

We recorded data in two types of experiments: (1) with both actuator arms pinned to the table (“isometric experiments”) and (2) while manually closing and opening the actuator (“work-loop experiments”). In the isometric experiments, we characterize the static torque–pressure relationship. The work-loop experiments allow us extracting the mechanism's friction and output efficiency in movement.

### Isometric experiments

We designed the actuator with a single shell and piston, with a constant shell- and piston-radius, therefore ensuring a constant piston-pressure area and effective lever arm. Consequently, we expect the actuator to produce torque linearly dependent on the pressure and independent of the working angle [Eq. (1)]. The data support our expectation, and the pressure–torque relationship fits a linear model for all joint angles from 0° to 100° (Fig. 7), with an average deviation of:

(6) $$\frac{1}{n}\sum_{i=1}^{n}\left|\frac{M_{recorded}-M_{calculated}}{M_{recorded}}\right|=1.3\%.$$

We tested our actuator up to p=50 kPa (0.5 bar), where it produced the maximum measured isometric torque of $M_{output}=7.3Nm$. This torque corresponds to the product of active pressure area and lever arm of $Ar=1.46\cdot 10^{-4}m^{3}$. The simplest model includes an active pressure area equal to the piston base area of 3044 mm^2^, and a 39 mm lever arm of its geometric center. This model estimates a torque output of 5.9 Nm at 50 kPa, or 80% of the measured torque (Table 1, Model 1). However, the rotary-rolling diaphragm's edge also acts as active area and contributes to the torque. If we include half of the rotary-rolling diaphragm gap as active pressure area and its lever arm, the modeled torque is $M_{model3}=$3557 mm^2^· 38.9 mm ·50 kPa = 6.9 Nm (Table 1, Model 3), or 95% of the measured torque. We derived the modeled torque from the actuator's ideal CAD model. In reality, 3D printing has limited accuracy, and printed parts tend to be slightly larger than their model, which would effectively increase the pressure active area. We suggest the 3D printer's limited accuracy as the likely explanation for the observed higher model torque values.

### Slower work-loop experiments

An example for a work-loop experiment with p=10.25 kPa chamber pressure and 8°s^−1^ angular speed is shown in Figure 8a. The curve starts at the maximum actuator angle (100°) and follows it in counterclockwise direction until the joint is closed at 0°. The opening stroke finishes the loop as the lower curve. Five recordings are averaged (solid line), and the shaded area indicates the 95% confidence interval. At 10.25 kPa averaged pressure, the actuator outputs an equivalent torque of *M*_output_ = 1.47 Nm torque (horizontal line, dashed). The mechanism's friction during the stroke creates a torque $M_{friction}$, visible as the offset from the average torque, forming the boundaries of the hysteresis loop. Here, friction leads to an average torque loss of *M*_friction_ (10.25 kPa) = 0.078 Nm, equivalent to a torque efficiency of $1-\frac{M_{friction}}{M_{output}}=1-\frac{0.078Nm}{1.47Nm}=94.7\%$. This mechanical efficiency only includes mechanical sources of losses, that is, friction. We chose to omit other sources of losses, such as from generating the pressure, and thermodynamic compression and decompression effects, as they are (1) inherent to all fluidic actuators and (2) largely depend on the chosen setup and operating speed and thus further complicate comparison.

Over the full pressure range, the counter-torque increases at an average rate of 5.2 mNm/kPa. The relative torque friction loss reaches a minimum of 3%, at 30 kPa chamber pressure. The actuator's average torque efficiency is 95% (Table 3, 1–50 kPa). This average torque efficiency is calculated by integrating its curve (Fig. 8b, green dashed) and dividing it by the interval length, to avoid the influence of the irregular sample spacing.

**Table 3.** Calculated Efficiency = 1 – [M(friction)/M(output)]

	Efficiency (%)
Average of all data	95.0
Maximum efficiency	97.1

The average efficiency is calculated by integrating the curve (Fig. 8b, green dashed) and dividing it by the interval length, avoiding the influence of irregular sample spacing.

Rolling diaphragms in piston–cylinder actuators transfer forces symmetrically between shell and piston, unlike our diaphragm. Forces exerted by the pressurized fluid produce the desired torque, but potentially also an additional component directed at the bridge. At high pressures (40–50 kPa) and large opening angles (above 60° joint angle), we observed the actuators' bridge slightly bending outward (Figs. 8c and 9). We assume that the bridge's deflection leads to a misalignment between the piston and the shell, continuously reducing the gap size and increasing the diaphragms rolling resistance. A stiffer bridge design or material would minimize deflection. The rate of observed friction increase is lower than the torque increase (5.2 vs. 146 mNm/kPa), and with a stiffer bridge, we expect to increase efficiency at higher pressures.

We observed a small change in output torque, around 30° joint angle and all chamber pressures. We traced it back to fabrication, where we unintentionally reinforced the diaphragm at one spot. This slightly thicker patch unrolls at 30° joint angle and produces the observed brief change in torque (Fig. 8a). No other joint angles are affected. Our observation emphasizes how important a uniform diaphragm thickness is.

### Fast work-loop experiments

We tested the actuator at 10 kPa target chamber pressure and 100°s^−1^ target (average) speed (105.1°s^−1^ average), that is, 12.5 × faster than previous experiments. We observe a chamber pressure of 8.8 ± 5.6 kPa (Fig. 8d). We also calculated the equivalent output torque $M_{equiv}\left(8.8kPa\right)=1.31Nm$, with an average friction torque of $M_{friction}=$0.056 Nm, equivalent to a mechanical torque efficiency of 95.7%.

The computational correction for differences in chamber pressure can compensate for torque changes caused by moderate pressure differences (Fig. 8b, c). At 8°s^−1^, the chamber pressure varied on average by ±10% (10.25 ± 1.03 kPa). We point out that it cannot compensate for additional effects on the diaphragm's unrolling behavior. These effects might become prominent when the pressure deviates much between opening and closing, as it is the case for the 100°s^−1^ joint speed experiments (±64%).

In sum, our results confirm that our actuator is capable of fast dynamic movement while still retaining its linear and low friction qualities.

## Conclusions

We demonstrated a new rotary-rolling diaphragm actuator, which works with high mechanical efficiency. On average, less than 5% output torque is lost by friction torque. The presented design achieves a linear pressure–torque relationship throughout a stroke of 100°. The sealed design can be the basis for a highly stiff actuator using an incompressible fluid, or for a compliant actuator by using air. The actuator is built from few major parts: the diaphragm, shell, and the piston/bridge.

The rotary-rolling diaphragm actuator is not as straightforward as rolling diaphragms used in telescopic actuators, and not commercially available. But it is customizable and can be implemented with easily available tools and materials: a 3D printer, a sewing machine, silicone rubber, a microfiber cloth, and a set of hand tools. Its compact design can quickly be adapted for robotic structures (Supplementary Video S1*).

We emphasize the direction-dependent stretchability of the reinforced rotary-rolling diaphragm, which avoids losses from elastic deformation, bulging, stiction, or Coulomb friction. A small one-directional stretchability is, however, required to roll the diaphragm between piston and shell radius. An industrially made, 3D-shaped core could be woven into a single piece, uniformly thin, with ideal fiber alignment, and should reduce the rolling friction even further.

Because of the soft 3D-printed material, we limited the pressure to 50 kPa (7.3 Nm torque, Fig. 5). We chose 3D printing for its workflow and availability. A stronger material and stiffer design would resist higher pressures and lead to higher torque, power density, and efficiency. A circular, 12 mm diameter diaphragm sample withstood more than 16 × the actuator's maximum pressure (800 kPa) in a dedicated test. If shell and piston were made from aluminum (45 × higher Young's modulus than printed ABS^48^), the rotary-rolling diaphragm actuator could produce at least 16×7.3Nm=117 Nm torque.

Partially soft shells could replace the rotary-rolling diaphragm actuator's outer shell pieces, according to the biological model. The spider's exoskeleton is made of elastic chitin. It has varying stiffness and softness, such as at the spider's opisthosoma and the spider's joint membrane, which acts as the outer wall after unrolling.^49^

Here, we focused on the mechanical efficiency of the rotary-rolling diaphragm actuator; however, pneumatically actuated systems exhibit additional losses compressing and decompressing air. The system's model animal, the spider, applies an incompressible fluid, and we plan to pressurize future actuator versions similarly.

The demonstrated rotary-rolling diaphragm actuator is a single-action actuator, for joint opening (Supplementary Video S1). Like telescopically acting rolling diaphragm actuators, the diaphragm requires a positive pressure difference to function properly.^50^ Existing double-action implementations of rolling diaphragm actuators should be directly transferable.^46,51^

Our rotary-rolling diaphragm actuator is compact and relatively lightweight, customizable, with low friction at low and high output torques, and 95% mechanical efficiency (Table 3). Its torque linearity throughout the stroke will simplify its control; the output torque can directly be calculated from the instantaneous chamber pressure and requires no inverse mapping of nonlinear pressure–torque relationships. And without stiction and Coulomb friction at slow (8°s^−1^) and fast (100°s^−1^) speed, the actuator should perform well in torque control applications, where linear output characteristics matter.^9^ The actuator is a fitting candidate for mobile systems such as legged robots. It could be applied as an air-spring or part of a fluidic damper.^52–55^ Its metal pins and fasteners can be replaced with plastic ones, for applications in strong magnetic fields, such as magnetic resonance imaging.^50^

## Supplementary Material

Supplemental data

## Notes

^*^
https://youtu.be/Cf8FyYw099g

## References

[1] De Volder M, Reynaerts D. Development of a hybrid ferrofluid seal technology for miniature pneumatic and hydraulic actuators. Sensors Actuat A Phys 2009;152:234–240.

[2] Raibert MH, Brown HB Jr, Murthy SS. Machines that walk. In: Brady M, Gerhardt LA, Davidson HF (eds). Robotics and Artificial Intelligence. Berlin, Heidelberg: Springer, 1984, pp. 345–364.

[3] Semini C, Tsagarakis N, Vanderborght B, *et al*. HyQ—Hydraulically actuated quadruped robot: Hopping leg prototype. In: 2008 2nd IEEE RAS & EMBS International Conference on Biomedical Robotics and Biomechatronics, Scottsdale, AZ, October 19–22, 2008, pp. 593–599. IEEE.

[4] Dejager EJ. Vane-type actuator. January 12, 1971. US Patent 3,554,096.

[5] Xia J, Durfee WK. Modeling of tiny hydraulic cylinders. In: 52nd National Conference of Fluid Power, March 23–25, 2011, pp. 1–5.

[6] Agarwal NK, Lawson CP. A practical method to account for seal friction in aircraft hydraulic actuator preliminary design. Proc Inst Mech Eng Part G J Aerosp Eng 2017;231:941–950.

[7] Team P. Parker: Rack and Pinion or Vane Actuators—Pros and Cons. Library Catalog: blog.parker.com (accessed December 20, 2020).

[8] Nikas G, Burridge G, Sayles R. Modelling and optimization of rotary vane seals. Proc Inst Mech Eng Part J J Eng Tribol 2007;221:699–715.

[9] Whitney JP, Glisson MF, Brockmeyer EL, *et al*. A low-friction passive fluid transmission and fluid-tendon soft actuator. In: 2014 IEEE/RSJ International Conference on Intelligent Robots and Systems, September 14–18, 2014, pp. 2801–2808. IEEE.

[10] Meier P, Lang M, OberthÃijr S. Reiterated tension testing of silicone elastomer. Plast Rubb Compos 2005;34:372–377.

[11] Dietrich J, Meier P, Oberthur S, et al. Development of a peristaltically actuated device for the minimal invasive surgery with a haptic sensor array. In: Micro- and Nanostructures of Biological Systems, Halle, Germany: Shaker-Verlag, 2004.

[12] Gillespie MT, Best CM, Killpack MD. Simultaneous position and stiffness control for an inflatable soft robot. In: 2016 IEEE International Conference on Robotics and Automation (ICRA), May 16–21, 2016, pp. 1095–1101.

[13] Shepherd RF, Ilievski F, Choi W, *et al.* Multigait soft robot. Proc Natl Acad Sci USA 2011;108:20400–20403. 22123978 10.1073/pnas.1116564108 PMC3251082

[14] Chou C-P, Hannaford B. Measurement and modeling of McKibben pneumatic artificial muscles. IEEE Trans Robot Autom 1996;12:90–102.

[15] Paez L, Agarwal G, Paik J. Design and analysis of a soft pneumatic actuator with origami shell reinforcement. Soft Robot 2016;3:109–119.

[16] Nemiroski A, Shevchenko YY, Stokes AA, *et al.* Arthrobots. Soft Robot 2017;4:183–190. 29182080 10.1089/soro.2016.0043

[17] Tu Q, Wang Y, Yue D, *et al.* Analysis on the impact factors for the pulling force of the McKibben pneumatic artificial muscle by a FEM model. J Robot 2020;2020:4681796.

[18] Sridar S, Majeika CJ, Schaffer P, *et al.* Hydro muscle—a novel soft fluidic actuator. In: 2016 IEEE International Conference on Robotics and Automation (ICRA), May 16–21, 2016, pp. 4014–4021.

[19] Chapman G. Versatility of hydraulic systems. J Exp Zoology 1975;194:249–269.

[20] Kang R, Branson DT, Zheng T, *et al.* Design, modeling and control of a pneumatically actuated manipulator inspired by biological continuum structures. Bioinspir Biomim 2013;8:036008. 23851387 10.1088/1748-3182/8/3/036008

[21] Beauzamy L, Nakayama N, Boudaoud A. Flowers under pressure: ins and outs of turgor regulation in development. Ann Botany 2014;114:1517–1533. 25288632 10.1093/aob/mcu187 PMC4204789

[22] Kropf C. Hydraulic system of locomotion. In: Nentwig W (ed.) Spider Ecophysiology. Berlin: Springer, 2013, pp. 43–56.

[23] Ellis C. The mechanism of extension in the legs of spiders. Biol Bull 1944;86:41–50.

[24] Parry DA, Brown RHJ. The hydraulic mechanism of the spider leg. J Exp Biol 1959;36:423–433.

[25] Manton S, Harding J. Hydrostatic pressure and leg extension in arthropods, with special reference to arachnids. J Nat Hist 1958;1:161–182.

[26] Parry DA. Spider leg-muscles and the autotomy mechanism. Q J Microsc Sci 1957;3:331–340.

[27] Weihmann T, Günther M, Blickhan R. Hydraulic leg extension is not necessarily the main drive in large spiders. J Exp Biol 2012;215:578–583. 22279064 10.1242/jeb.054585

[28] Faraji H, Tachella R, Hatton RL. Aiming and vaulting: spider inspired leaping for jumping robots. In: 2016 IEEE International Conference on Robotics and Automation (ICRA), May 16–21, 2016, pp. 2082–2087. IEEE.

[29] Landkammer S, Winter F, Schneider D, *et al.* Biomimetic spider leg joints: a review from biomechanical research to compliant robotic actuators. Robotics 2016;5:15.

[30] Menon C, Lira C. Spider-inspired embedded actuator for space applications. In: Proceedings of AISB'06: Adaptation in Artificial and Biological Systems, Bristol, UK, April 3–6, 2006.

[31] Spröwitz A, Göttler C, Sinha A, *et al*. Scalable pneumatic and tendon driven robotic joint inspired by jumping spiders. In: 2017 IEEE International Conference on Robotics and Automation (ICRA), May 29– June 03, 2017, pp. 64–70. IEEE.

[32] Munakata G, Zanini P, Titotto S. 3D fingerprint design proposal using spider movement mechanism and soft robotic technology. Res Biomed Eng 2020;36:361–368.

[33] Belforte G, Mattiazzo G, Mauro S, *et al.* Measurement of friction force in pneumatic cylinders. Tribotest 2003;10:33–48.

[34] Fujita T, Tokashiki LR, Kagawa T. Stick-slip motion in pneumatic cylinders driven by meter-out circuit. Proc JFPS Int Symp Fluid Power 1999;1999:131–136.

[35] Airpot C. Airpot Dashpot Comparison Table. Norwalk, CT: Airpot Corporation, 2020.

[36] Hashemi S, Durfee WK. Low friction, long-stroke rolling diaphragm cylinder for passive hydraulic rehabilitation robots. In: 2017 Design of Medical Devices Conference, American Society of Mechanical Engineers Digital Collection, 2017.

[37] Gruebele A, Frishman S, Cutkosky M. Long-stroke rolling diaphragm actuators for haptic display of forces in teleoperation. IEEE Robot Autom Lett, 2019;4:1478–1484.

[38] Diaphragm Design Guidebook. Amherst, New Hampshire 03031, USA, 4 ed., 2008.

[39] Diaphragm Design Manual. Weinheim, Deutschland, 2009.

[40] Technical Principles—Diaphragms. Weinheim, Deutschland, 1 ed., 2007.

[41] Tolley MT, Shepherd RF, Mosadegh B, *et al.* A resilient, untethered soft robot. Soft Robot 2014;1:213–223.

[42] Byrne O, Coulter F, Glynn M, *et al.* Additive manufacture of composite soft pneumatic actuators. Soft Robot 2018;5:726–736. 30148682 10.1089/soro.2018.0030

[43] Mullins L. Softening of rubber by deformation. Rubber Chem Technol 1969;42:339–362.

[44] Ogden RW, Roxburgh DG. A pseudoâĂŞelastic model for the Mullins effect in filled rubber. Proc Royal Soc London A Math Phys Eng Sci 1999;455:2861–2877.

[45] Chen Y, Wan F, Wu T, *et al.* Soft-rigid interaction mechanism towards a lobster-inspired hybrid actuator. J Micromech Microeng 2017;28:014007.

[46] Whitney JP, Chen T, Mars J, *et al*. A hybrid hydrostatic transmission and human-safe haptic telepresence robot. In: 2016 IEEE International Conference on Robotics and Automation (ICRA), May 16–21, 2016, pp. 690–695. IEEE.

[47] Cappello L, Galloway KC, Sanan S, *et al.* Exploiting textile mechanical anisotropy for fabric-based pneumatic actuators. Soft Robot 2018;5:662–674. 30024312 10.1089/soro.2017.0076

[48] Hoffmann T. Untersuchung der mechanischen Festigkeit von FDM Druckteilen. BS thesis, 2016.

[49] Barth FG. Microfiber reinforcement of an arthropod cuticle. Z Zellforsch Mikrosk Anat 1973;144:409–433. 4785981 10.1007/BF00307585

[50] Burkhard N, Frishman S, Gruebele A, *et al*. A rolling-diaphragm hydrostatic transmission for remote mr-guided needle insertion. In: 2017 IEEE International Conference on Robotics and Automation (ICRA), May 29– June 03, 2017, pp. 1148–1153. IEEE.

[51] Hashemi S, Sobojinski S, Durfee WK. Low-friction antagonist hydraulic transmission using long-stroke rolling diaphragm cylinders. In: ASME/BATH 2017 Symposium on Fluid Power and Motion Control, American Society of Mechanical Engineers, October 16–19, 2017, p. V001T01A073.

[52] Shen Z, Seipel J. A fundamental mechanism of legged locomotion with hip torque and leg damping. Bioinspirat Biomimet 2012;7:046010. 10.1088/1748-3182/7/4/046010 22989956

[53] Kalouche S. Goat: A legged robot with 3d agility and virtual compliance. In: 2017 IEEE/RSJ International Conference on Intelligent Robots and Systems (IROS), September 24–28, 2017, pp. 4110–4117. IEEE.

[54] Havoutis I, Semini C, Buchli J, *et al*. Quadrupedal trotting with active compliance. In: 2013 IEEE International Conference on Mechatronics (ICM), 2013, pp. 610–616. IEEE.

[55] Mo A, Izzi F, Haeufle DFB, *et al.* Effective viscous damping enables morphological computation in legged locomotion. Front Robot AI 2020;7. 33501277 10.3389/frobt.2020.00110 PMC7805837

